# Tracheal Adenoid Cystic Carcinoma Presented with Chronic Asthma Diagnosed by Bronchial Washing Cytology

**DOI:** 10.1155/2020/6543097

**Published:** 2020-01-16

**Authors:** Maryam Mohammadnia, Massood Hosseinzadeh, Perikala Vijayananda Kumar, Sahand Mohammadzadeh

**Affiliations:** Department of Pathology, Shiraz University of Medical Science, Shiraz, Iran

## Abstract

Adenoid cystic carcinoma is a tumor that mainly arises from salivary glands and is present rarely in airways with nonspecific symptoms. Diagnosis based on bronchial washing cytology is rarely reported because this tumor is usually lined by normal mucosa. A 35-year-old woman was referred to our center as a case of unresponsive asthma and hemoptysis for the past year. CT scan showed tracheal mass. Bronchoscopy was done followed by bronchial washing cytology and biopsy. Cytology smears revealed sheets and three-dimensional clusters of small cells, and some of them arranged around hyaline mucoid globules. Cell block and biopsy showed classic pathological findings of adenoid cystic carcinoma. Adenoid cystic carcinoma of the airways can be manifested with nonspecific symptoms and should be considered in the differential diagnosis of airway diseases and asthma. This tumor is rarely seen in the bronchial washing specimen. Characteristic cytological findings and using cell block preparation differentiate adenoid cystic carcinoma from other tumors.

## 1. Introduction

The incidence of primary tracheal tumors is infrequent. Adenoid cystic carcinoma (ACC) is a malignant tumor that mainly arises from major and minor salivary glands [1].

ACC of the lung arises from bronchial glands and mostly appears in the trachea and main bronchi [1, 2]. It is a rare disease and constitutes about 0.04–0.2% of the primary lung tumors [1]. In the past, this tumor was called bronchial adenoma, which is a wrong terminology because ACC is not a benign tumor [2].

There is limited epidemiologic data about ACC of the lung. Based on few studies, the mean age is 46 years (range of 22–73 years) and male to female incidence ratio is different in various studies [1, 2]. Symptoms are nonspecific, including cough, shortness of breath, and occasionally hemoptysis. Some patients might be misdiagnosed as asthma [1, 2]. Characteristic findings for ACC are not reported in CT scan or bronchoscopy, and thus the diagnosis is based on pathology.

Fine needle aspiration cytology is a routine method for sampling of ACC of salivary glands, but it is used rarely in airways. Bronchial washing and brushing is the popular cytology method in airways and the lung [3].

In the trachea and bronchi, ACC is usually covered by intact mucosa, and tumor cells are not found in bronchial cytology specimens. In rare cases, tumor invades to overlying mucosa, and tumor cells can be seen in bronchial washing or bronchial brushing specimens [4–7].

## 2. Case Report

A 35-year-old female presented with cough and shortness of breath for the past year. She was treated as asthma but did not respond, and hemoptysis appeared. CT scan was performed, and soft tissue mass arising from the posterior and lateral wall of the trachea with mediastinum extension was reported. The patient was undergoing bronchoscopy, and tracheal mass 3 cm above carina with invasion to the lateral wall of trachea was detected. Bronchial washing and biopsy were performed.

Alcohol-fixed smears from the bronchial washing specimen were prepared.

**Figure 1:**
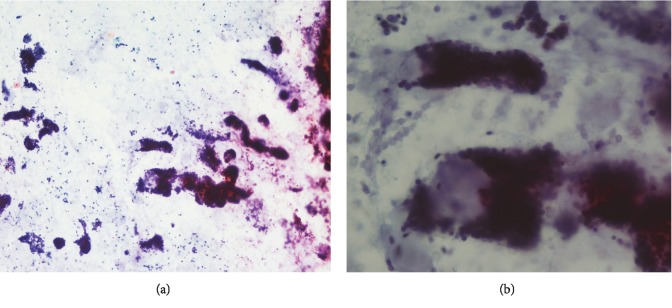
Cytology smears. (a) Sheets, three-dimensional clusters, and dispersed cells with hyaline materials (Papanicolaou stain, ×100). (b) Clusters of neoplastic cells surrounding hyaline globules (Papanicolaou stain, ×400).

Smears were stained with the modified Papanicolaou method. Cytology showed hypercellular smears composed of loosely cohesive sheets, three-dimensional clusters, and dispersed cells. Cells were relatively small and uniform with round nuclei, small nucleoli, and scant cytoplasm. Throughout the smears, acellular hyaline materials with globule formation in different sizes were seen, and some of them were enveloped by tumor cells (Figure 1).

**Figure 2:**
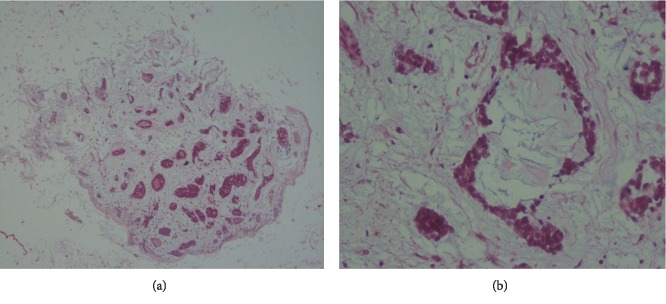
Cell block slide. (a) Tubular-like structure and cribriform pattern with acidophilic materials (H&E stain, ×100). (b) Small nests and strands of tumor cells (H&E stain, ×400).

A cell block was prepared using the thrombin method. In the cell block, nests and strands with tubular-like structures and a cribriform pattern containing homogenous acidophilic materials were seen (Figure 2).

**Figure 3:**
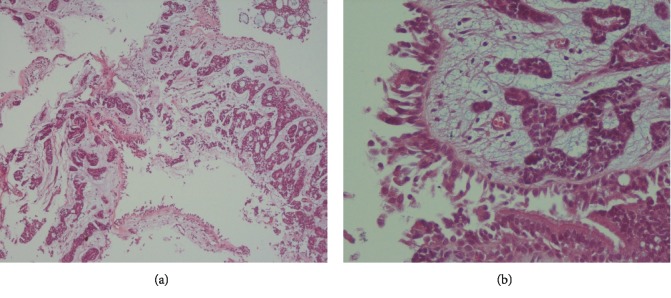
Endobronchial biopsy. (a) Bronchial mucosa contains infiltrative neoplastic lesions with tubular and cribriform patterns (H&E stain, ×100). (b) Tubular and cribriform structures of the neoplasm (H&E stain, ×400).

Biopsy revealed bronchial mucosa with infiltrative neoplastic lesions composed of tubular and cribriform structures with acidophilic materials (Figure 3).

The final diagnosis was adenoid cystic carcinoma. The patient was undergoing resection surgery, but unfortunately, complete resection was not possible, and then she was referred for radiotherapy.

## 3. Discussion

Adenoid cystic carcinoma of the lung is a rare disease and presents with nonspecific symptoms. It can be misdiagnosed as asthma [1]. Imaging and bronchoscopy cannot differentiate ACC from other tumors such as carcinoid tumor or squamous cell carcinoma, and final diagnosis was based on pathology. In most cases, bronchial cytology is negative because the tumor is covered by intact mucosa. Sometimes overlying mucosa is ulcerated and makes the diagnosis based on bronchial washing cytology possible [4–7]. Cytology smears show monolayer sheets, three-dimensional clusters, and many isolated cells. Tumor cells are small and uniform with round nuclei, small nucleoli, and minimal cytoplasm. In the background, acellular hyaline materials and spherical globules are seen. Some of these hyaline globules are surrounded by tumor cells; this finding is a significant diagnostic feature of ACC. Using the cell block shows characteristic features of ACC, including tubular structures and a cribriform pattern with acidophilic materials and facilitates the diagnosis of ACC.

Carcinoid tumor and small cell carcinoma are made of small cells; therefore, they are in differential diagnosis with ACC in cytology. Characteristic granular chromatin and absence of hyaline globules favor carcinoid tumor [8]. Features of small cell carcinoma, including granular chromatin, crush artifact, necrosis, and apoptotic bodies, are absent in ACC [9].

Tumor cells surrounding hyaline materials may be misdiagnosed as true glands, so well-differentiated adenocarcinoma enters in the differential diagnosis. Cytonuclear atypia of adenocarcinoma and absence of hyaline globules differentiates it from ACC [5].

## 4. Conclusion

The incidence of ACC in the trachea is infrequent, but it is crucial to consider this tumor if a patient does not respond to antiasthmatic therapy. In these cases, proper imaging with careful attention to cytologic criteria and preparing the cell block makes the accurate diagnosis possible.

## References

[1] Hu M.-M., Hu Y., He J.-B., Li B.-L. (2015). Primary adenoid cystic carcinoma of the lung: clinicopathological features, treatment and results. *Oncology Letters*.

[2] Bhattacharyya T., Bahl A., Kapoor R., Bal A., Das A., Sharma S. C. (2013). Primary adenoid cystic carcinoma of lung: a case report and review of the literature. *Journal of Cancer Research and Therapeutics*.

[3] Kundu R., Handa U., Punia R. S., Dass A., Saini V. (2018). Adenoid cystic carcinoma: a study of 19 cases of salivary and extra-salivary tumours diagnosed by fine needle aspiration cytology. *Diagnostic Cytopathology*.

[4] Daneshbod Y., Modjtahedi E., Atefi S., Bedayat G. R., Daneshbod K. (2007). Exfoliative cytologic findings of primary pulmonary adenoid cystic carcinoma. *Acta Cytologica*.

[5] Chuah K. L., Lim K. H., Koh M. S., Tan H. W., Yap W. M. (2007). Diagnosis of adenoid cystic carcinoma of the lung by bronchial brushing. *Acta Cytologica*.

[6] Buchanan A. J., Fauck R., Gupta R. K. (1988). Cytologic diagnosis of adenoid cystic carcinoma in tracheal wash specimens. *Diagnostic Cytopathology*.

[7] Chen K. T. K. (1996). Exfoliative cytology of tracheobronchial adenoid cystic carcinoma. *Diagnostic Cytopathology*.

[8] Aron M., Kapila K., Verma K. (2004). Carcinoid tumors of the lung: a diagnostic challenge in bronchial washings. *Diagnostic Cytopathology*.

[9] Sturgis C. D., Nassar D. L., D’Antonio J. A., Raab S. S. (2000). Cytologic features useful for distinguishing small cell from non-small cell carcinoma in bronchial brush and wash specimens. *American Journal of Clinical Pathology*.

